# The effect of public hospital closure on the death of long-term inpatients in Korea

**DOI:** 10.4178/epih.e2024022

**Published:** 2024-01-17

**Authors:** Taeuk Kang, Minsung Sohn, Changwoo Shon

**Affiliations:** 1 Health and Wellness College, Sungshin Women’s University Woonjung Green Campus, Seoul, Korea; 2 Division of Health Care Science, The Cyber University of Korea, Seoul, Korea; 3 Graduate School of Public Health, Inje University, Busan, Korea

**Keywords:** Public health, Health services accessibility, Socioeconomic factors, Public hospitals

## Abstract

**OBJECTIVES:**

This study aimed to examine the changes in health outcomes and the patterns of medical institution utilization among patients with long-term stays in public hospitals following the closure of a public medical center. It also sought to present a proposal regarding the role of public hospitals in countries with healthcare systems predominantly driven by private entities, such as Korea.

**METHODS:**

To assess the impact of a public healthcare institution closure on health outcomes in a specific region, we utilized nationally representative health insurance claims data. A retrospective cohort study was conducted for this analysis.

**RESULTS:**

An analysis of the medical utilization patterns of patients after the closure of Jinju Medical Center showed that 67.4% of the total medical usage was redirected to long-term care hospitals. This figure is notably high in comparison to the 20% utilization rate of nursing hospitals observed among patients from other medical facilities. These results indicate that former patients of Jinju Medical Center may have experienced limitations in accessing necessary medical services beyond nursing care. After accounting for relevant mortality factors, the analysis showed that the mortality rate in closed public hospitals was 2.47 (95% confidence interval, 0.85 to 0.96) times higher than in private hospitals.

**CONCLUSIONS:**

The closure of public medical institutions has resulted in unmet healthcare needs, and an observed association was observed with increased mortality rates. It is essential to define the role and objectives of public medical institutions, taking into account the distribution of healthcare resources and the conditions of the population.

## INTRODUCTION

The roles and responsibilities of public hospitals differ based on the healthcare system and ideological beliefs of a particular country. In Korea, the central government sets the direction of healthcare policies, and local governments operate regional medical centers as public hospitals to implement healthcare policies [1]. Although the Korean healthcare system is primarily publicly financed—based on social insurance—the presence of public hospitals is relatively minor, with private hospitals comprising 90.3% of all hospital beds [2,3]. The Public Health and Medical Services Act specifies the duties and obligations of public hospitals to maintain the public nature of healthcare in Korea. These responsibilities include providing medical services to vulnerable populations and underserved areas, managing and preventing both infectious and non-infectious diseases at the local government level, and offering disaster medical care [4]. This legislation was enacted to ensure a baseline level of public service through a limited number of public hospitals.

Against this backdrop, we focused on the closure of Jinju Medical Center—a notable public hospital with around 250 beds located in Gyeongsangnam Province in Korea—which was announced in 2013. At that time, the governor of Gyeongsangnam Province held the view that efficiency was just as crucial as the public service aspect in the operation of public hospitals. The patient demographic at Jinju Medical Center skewed towards low-income individuals, and the proportion of labor costs for workers was also high. As a result, the financial health of the hospital continued to decline [5]. To make matters worse, Jinju Medical Center had accumulated a deficit of approximately 28 billion Korean won (KRW), with an annual shortfall of 4 billion KRW to 6 billion KRW [6]. Consequently, the governor decided to close the hospital.

In 2013, Jinju City had 2.9 doctors per 1,000 people and 14.7 hospital beds per 1,000 people, figures that exceeded the Korean averages of 2.6 doctors and 12.3 beds per 1,000 people, respectively [7]. In addition, as many patients in public hospitals were long-term inpatients, often admitted for “social admission”—hospitalization without an acute medical condition—the closure of the public hospital was not anticipated to significantly impact acute care patients. Nevertheless, to mitigate potential harm to inpatients, it was crucial that those discharged from Jinju Medical Center were effectively connected with the appropriate community resources. Unfortunately, this critical aspect appeared to have been inadequately addressed. Indeed, without proper case management and community care based on available health and welfare resources post-discharge, patients are at an increased risk of developing further disabilities. This risk is even more pronounced among lower-income groups [8]. The closure of public hospitals in Korea is extremely rare, and the shutdown of Jinju Medical Center is unique as it was the only general hospital-level public hospital in the region to be closed.

Therefore, this study aimed to examine the changes in health outcomes and healthcare facility utilization patterns among patients who were long-term hospitalized in public hospitals after the Jinju Medical Center closed. It also sought to highlight the role and significance of public hospitals within healthcare systems that are predominantly private, as is the case in Korea. The specific objectives of the study were as follows: First, to analyze where inpatients were transferred following the closure of public hospitals. Second, to assess the one-year mortality rate of these inpatients after the closure. Third, to investigate mortality differences among patients who were admitted to the now-closed public hospitals, considering variables such as gender, age, income, and pre-existing medical conditions. Fourth, to elucidate the significance and implications of public hospitals in the context of the healthcare system.

## MATERIALS AND METHODS

### Study design and data sources

This study investigated the potential health hazards associated with the closure of a public medical institution that had 250 beds, situated in an area with a population of approximately 350,000 people. The study utilized data from health insurance claims that encompassed the entire population. While the National Health Insurance (NHI) claims database may not contain information on individual socioeconomic factors, it offers the benefit of providing comprehensive data on medical utilization collected by healthcare institutions. The study was conducted in 2 stages. In the first stage, medical institutions were chosen as research subjects based on their type of establishment and geographic location. All 37 public hospitals across the country were included, with the exception of specialized institutions such as military or police hospitals. In addition, 25 private hospitals located in the same administrative region as the now-closed Jinju Medical Center were selected. In the second stage, individuals who were hospitalized between June 2012 and December 2012 were identified from the 63 medical institutions initially selected. A total of 353,650 individuals were included in the study, with 192,902 from the 37 public hospitals and 160,748 from the 25 private hospitals. The final study participants were those who had been hospitalized for 90 days or more at the same medical institution during the period from June 2012 to December 2012. From this group, 9,522 patients were selected, which included 259 patients from the Jinju Medical Center, 6,488 from other public hospitals, and 2,775 from private hospitals.

### Measurement

#### Individual variables

This study analyzed socio-demographic variables including gender, age, disability, type of health insurance, and premium decile, as well as health-related variables such as diagnosis, Charlson comorbidity index (CCI), and mortality status. The NHI claims database provides essential information for insurance claims, encompassing demographic details like gender, age, and region; International Classification of Diseases, 10th revision (ICD-10) diagnosis codes; medical services rendered; and medical costs. However, it lacks variables such as individual income and education level, which are crucial for socio-demographic research.

#### Outcome variables

Given our research objectives, our initial research effort focused on identifying the medical institutions that hospital inmates turned to following the closure of a public hospital. We categorized these institutions into seven groups and chose their utilization rates as an outcome measure. Subsequently, our second and third objectives involved comparing the health outcomes of former hospital inpatients with those of patients at other medical institutions after the closure. For this comparison, we selected mortality as the outcome measure.

### Empirical methods

Logistic regression analysis was employed if there was a statistically significant difference in mortality rates between long-term patients at Jinju Hospital and those at other medical facilities. The dependent variable was whether the patient died within one year of discharge. The primary study variable was the type of hospital (Jinju Hospital, public hospital, private hospital), with control variables including gender, age, income quintile, and CCI. In relation to the dependent variable of mortality, 12 patients who died within 1 month of the closure of Jinju Hospital were excluded from the final analysis to mitigate the potential overestimation of the hospital’s closure impact. Furthermore, this study tackled the issue of selection bias by employing the propensity score matching method. Optimal full matching was utilized, taking into account potential confounders such as gender, age, income percentile, ICD criteria, and CCI. Optimal full matching organizes research subjects with similar propensity scores from both the treatment and control groups into the same stratum, thereby stratifying the entire dataset. During this process, subjects are paired into multiple strata, and within each stratum, matches are made based on the proportionate sample sizes of the treatment and control groups. As a result, from the initial pool of 9,522 study participants, 1,466 were finally selected.

## RESULTS

### Characteristics of long-term inpatients in healthcare facilities

Upon examining the characteristics of long-term hospitalized patients by hospital type, it was found that 7.0% of patients at Jinju Medical Center had been hospitalized for more than 90 days, compared to 2.4% at public hospitals and 1.2% at private hospitals. The incidence of long-term hospitalization at Jinju Medical Center was approximately 3-5 times higher than at other types of hospitals. The average length of stay was 30.8 days at Jinju Medical Center, 16.9 days at public hospitals, and 11.6 days at private medical institutions, indicating that patients at Jinju Medical Center had longer stays than those at other medical facilities. To further examine patient characteristics, we stratified the data by gender, age, disability grade, and primary diagnosis (Table 1). This analysis showed that 54.7% of patients at Jinju Medical Center were aged 70 years or older, a figure about twice as high as that in private hospitals (21.6%) and other public hospitals (27.1%). In terms of disease characteristics, there was a high proportion of patients with neurological diseases.

### Changes in healthcare utilization patterns among primary users after the closure of a public healthcare institution

#### Analysis of medical utilization of inpatients from closed medical institutions

To identify changes in medical utilization patterns among patients who had been hospitalized for extended periods at Jinju Medical Center following its closure, we analyzed the medical utilization rates by type of medical institution for these patients from May 2013 to April 2014. We used a normalized total medical utilization amount, setting it to 100 as a reference point. The types of medical institutions included in the study were general hospitals, hospitals, nursing homes, mental healthcare institutions, dental clinics, public health centers, and oriental medicine hospitals. For the purpose of comparison, we selected data from a diverse range of healthcare facilities: 38 public hospitals across the nation, 25 private hospitals in the Gyeongsangnam Province region where Jinju Medical Center was located, and 3 private hospitals in the same locality with a similar number of beds to Jinju Medical Center (Table 2).

#### Analysis of inpatient behavior of patients admitted to the closed medical institution

Next, we analyzed the patterns of medical admissions for long-term patients at Jinju Medical Center over the year following its closure. Our analysis focused on the average number of admissions, the average length of stay per admission, and the number of admissions through the emergency room, with these variables categorized by income level. The results indicated that emergency room admissions were 2.1 times more frequent for medical aid recipients and 3.6 times more frequent for individuals in the first income quintile compared to 0.6 times for those in the fifth income quintile. Furthermore, the low-income group, comprising Medical Aid recipients and those in the first income quintile, had an average of 6.3 admissions and discharges within the year. In contrast, as income level increased, the frequency of admissions and discharges decreased, and there was a trend toward longer hospital stays (Table 3).

### Analysis of the impact of public medical institution closure on the health of hospital patients

#### Analysis of mortality after closure of public healthcare institutions

To the impact of a public medical institution’s closure on the health status of its users, we conducted a comparative analysis of the total number of deaths at one month and 1-year post-closure. Our findings indicated no significant difference in the 1-year mortality rate between the Jinju Medical Center and neighboring private hospitals. Nevertheless, there was a slight increase in the death rate within the first month following the closure (Table 4).

#### Analysis of mortality rate comparison after closure of public healthcare institutions

Among the 259 patients who were transferred to other healthcare facilities for primary treatment following the closure of Jinju Medical Center, 23.6% died within one year, with 4.6% dying within the first month. While the abrupt closure may have contributed to the decline in health among critically ill patients, a more detailed analysis is required to understand the mortality rate difference, which ranges from 5.5% to 9.8% when compared with the control group. This analysis should consider the potential effects of the transfer process over the course of a year. A logistic regression analysis, with mortality as the dependent variable, was conducted on the selected sample. The results indicated that patients who had been hospitalized long-term in the now-closed public healthcare institution faced a higher risk of death than those in the control group. Specifically, the mortality risk was 2.4 times higher when compared with private hospitals in the same administrative area, and 1.14 times higher when compared with private hospitals in nearby regions. In terms of economic status, individuals in the highest income quintile had a 2.24 times greater risk of death compared to those receiving Medical Aid. This was followed by a 1.34 times higher risk for individuals in the lowest income quintile and a 1.28 times higher risk for those in the second income quintile. Overall, the data suggest that individuals in lower-income brackets experienced higher mortality rates (Table 5).

## DISCUSSION

The present analysis of the effects of the closure of a public medical institution with 250 beds highlighted the potential obstacles to accessing medical care faced by vulnerable and low-income groups in rural subregions that lack public healthcare. This study investigated the impact of a public medical institution closure on healthcare utilization and the health status of local residents.

The primary findings of this study are as follows: First, an analysis of the patient characteristics at Jinju Medical Center revealed that the average length of stay was 1.8 times longer than that at other public hospitals and nearby private medical institutions. Furthermore, the incidence of long-term hospitalizations exceeding 90 days was three times to five times greater than at other public or private hospitals. An examination of individual characteristics showed that 54.7% of the participants were elderly, aged over 70 years, which is approximately double the rate found in other public and private hospitals. Additionally, there was a higher proportion of patients with neurological diseases at Jinju Medical Center compared to other disease groups.

Second, after the closure of the Jinju Medical Center, 67.4% of the total medical usage was transferred to long-term care hospitals. This shift was notably higher than the 54.7% observed for other types of institutions. The tendency to move patients to long-term care facilities may be driven by the higher incidence of neuropsychiatric diseases and other specific disease characteristics, indicating that patients are sometimes placed in long-term care even when hospitalization may not be medically necessary, due to a lack of alternative options. An analysis of income levels showed that participants from the high-income bracket who had previously utilized medical centers were more likely to transition to long-term care hospitals. This suggests that following the closure of their local medical centers, low-income groups may struggle to access medical services and could face unmet healthcare needs due to insufficient information [9]. From the perspective of healthcare providers, the influx of low-income patients may not be financially lucrative. Additionally, the community’s healthcare demands have been strained by the closure of public hospitals, leading to situations where some individuals have been unable to receive treatment because they are unwilling or unable to switch to alternative medical institutions.

Third, regarding the health outcomes of local residents, a notable disparity was observed in the overall mortality rate between Jinju Medical Center and neighboring private hospitals in the year following the center’s closure. The mortality rate was comparatively high within one year after Jinju Medical Center ceased operations. Factors that contributed to the increased mortality included long-term hospitalized patients, male patients, and those receiving medical assistance from lower-income brackets. A shift in healthcare utilization, such as unmet care needs, precipitated immediate health consequences [9]. This can be understood within the context of the recent coronavirus disease 2019 (COVID-19) pandemic, in which limited access to medical care resulted in a significant increase in the death rate among residents in need of treatment in various communities [10].

In this analysis of the unprecedented closure of public hospitals in Korea, we found that the shutdown of a public hospital in Korea had a profound impact, posing a significant health threat to low-income patients and disrupting the regional healthcare delivery system. In light of these findings, we propose several policy recommendations. First, there is a need to revise the performance assessment criteria for Korean public hospitals to better reflect their functions and roles. Currently, these hospitals are evaluated and funded based on factors such as healthcare service quality, the provision of specialized regional healthcare services, efficient administration, and responsible management. However, the primary reason for the closure of the Jinju Medical Center was its accumulated financial deficit and operational inefficiencies. Given the critical role that public hospitals serve in their communities, it may be beneficial to adjust the assessment criteria to reflect the unique medical and socioeconomic conditions of each community, rather than applying a one-size-fits-all evaluation model to all public hospitals [11]. In light of the emerging role of public hospitals in providing treatment to low-income patients, the concept of a “good deficit” has gained prominence, considering their public interest functions [12,13]. The financial shortfall at the Jinju Medical Center was largely due to low hospitalization revenue, with a substantial number of inpatients being low-income individuals in need of long-term care. Evaluations of public healthcare institutions should not solely focus on profitability but also on their ability to provide necessary treatment and essential medical services to the region. The roles and objectives of public medical institutions must be customized to the distribution of medical resources and the demographic conditions of each region. When assessing the efficiency of public health institutions, considerations should extend beyond allocative and technical efficiency to include social efficiency. This broader perspective ensures equitable access to medical services, also known as health equity [14].

Second, to establish the functions of public hospitals, it is necessary to clarify their concepts and roles. Public hospitals should concentrate on delivering essential medical services such as emergency care, infectious disease control, hospice care, and rehabilitation. These services are often less profitable for private medical institutions but are crucial for public health. Additionally, public hospitals should serve the needs of vulnerable populations. Rather than competing with other hospitals in the community, public hospitals should focus on providing care that aligns with policy needs. For example, services such as obstetrics, infectious diseases, low-demand specialties, specialized diseases or injuries, and care for individuals with disabilities often require additional resources. It is therefore advantageous for public hospitals to address these areas. The World Health Organization defines the role of hospitals as providing high-quality, comprehensive, and appropriate medical care, regardless of whether they are publicly or privately owned [15,16]. Furthermore, when setting the strategic direction of public hospitals, it is essential to actively involve the local community. This ensures that the services provided meet the needs of local residents and that policy directions are driven by demand [17].

Finally, the closure of public hospitals in a community should be approached with caution due to the potential impact on the community’s ability to respond to public health crises. When an infectious disease outbreak occurs on a large scale in a short period, as seen during the COVID-19 pandemic, there is a risk of a medical surge overwhelming the system. This can happen in the absence of effective management by community-based public hospitals. Such crises pose a significant threat to health, potentially leaving residents without access to adequate healthcare during critical times [18].

This study has the following limitations. First, although the study found a higher mortality rate among long-term inpatients at the Jinju Medical Center than in the control group, it cannot definitively establish a causal link between the closure of Jinju Medical Center and these deaths based solely on the observed restrictions in medical usage. While the study investigated the impact of income class on medical usage restrictions, including readmission rates, length of hospital stay, and emergency room visits, these findings cannot be exclusively attributed to the closure of Jinju Medical Center. Second, the study presumed that the characteristics of public medical center users in Korea would be consistent by including all public hospitals except for specialized ones. Nevertheless, Jinju Medical Center had a notably higher proportion of elderly and low-income patients, with a greater incidence of dementia and cerebrovascular disease compared to other public institutions. These conditions make physical activity and home caregiving particularly difficult. To address potential patient-related biases, the study employed propensity score matching and regression analysis. Third, although using administrative data provided an advantage in this study, it is crucial to acknowledge the limitations of such data when assessing individual characteristics that influence healthcare utilization. The model included only gender, age, health insurance income quintile, disability status, and pre-existing comorbidities (CCI) as factors affecting healthcare utilization.

This study explored the impact of a public hospital closure on local access to healthcare and the subsequent health outcomes for community members. Further research is needed to examine the intricacies of the closure process, with a particular focus on integrating the viewpoints and concerns of those affected by the shutdown of hospitals. Given the difficulty in generalizing the establishment, operation, and purpose of public hospitals due to diverse cultural, social, and economic factors, this study suggests a comparative analysis to examine the variations in decision-making processes regarding hospital closures in different countries. Comparative studies of this nature could yield important insights into the myriad factors that affect healthcare infrastructure and access on a global scale.

## Figures and Tables

**Table 1.** Comparison of the characteristics of 90-day long-term hospitalized patients according to their place of care

Risk factors	Public hospitals	Private hospitals	Jinju Hospital
Gender			
Men	1,129 (60.3)	302 (63.2)	30 (56.6)
Women	745 (39.8)	176 (36.8)	23 (43.4)
Age (yr)			
<40	136 (7.2)	39 (8.1)	0 (0.0)
40-49	377 (20.1)	110 (23.0)	4 (7.6)
50-59	552 (29.5)	161 (33.7)	15 (28.3)
60-69	302 (16.1)	65 (13.6)	5 (9.4)
70-79	283 (15.1)	65 (13.6)	14 (26.4)
≥80	224 (12.0)	38 (8.0)	15 (28.3)
Disability			
Non-disabled	837 (44.7)	220 (46.0)	31 (58.5)
Grade 1	209 (11.2)	66 (13.8)	8 (15.1)
Grade 2	377 (20.1)	94 (19.7)	6 (11.3)
Grade 3	451 (24.0)	98 (20.5)	8 (15.1)
Classification of diseases (ICD-10 code)			
Mental and behavioral disorders (F00-F99)	196,516 (25.5)	54,417 (16.6)	13,371 (25.3)
Nervous system disease (G00-G99)	44,545 (5.8)	28,936 (8.8)	10,929 (20.6)
Circulatory system disease (I00-I99)	68,221 (8.9)	51,880 (15.8)	7,161 (13.5)
Neoplasm (C00-D48)	63,113 (8.2)	52,400 (16.0)	4,409 (8.3)
Diseases of the musculoskeletal system and connective tissue (M00-M99)	54,292 (7.1)	22,733 (6.9)	3,751 (7.1)

Values are presented as number (%). ICD-10, International Classification of Diseases, 10th revision.

**Table 2.** Analyzing post-discharge healthcare utilization among long-stay patients (May-December 2013)

Hospital type	Public hospitals (n=38)		Private hospitals located in Jinju (n=25)^1^	
	Jinju Medical Center (n=1)	Other public hospitals (n=37)	Group 1 (n=22)	Group 2 (n=3)
General hospital	6.5	59.7	60.4	48.7
Hospital	19.6	11.4	11.0	16.5
Nursing hospital	67.4	19.5	19.4	24.4
Mental nursing hospital	4.8	6.3	5.9	6.9
Dental hospital	0.2	0.4	0.4	0.7
Public health	0.6	0.2	0.3	0.2
Korean medicine hospital	0.9	2.5	2.6	2.6

1 Group 1: 22 Private hospitals located within the Jinju City; Group 2: 3 Private hospitals with a similar number of beds to Jinju Medical Center.

**Table 3.** Analysis of long-term inpatient healthcare utilization 1 year after the closure of Jinju Medical Center (comparative analysis by income level)

Variables	Average no. of hospitalizations	Average hospitalization days	Average no. of hospitalizations via the emergency room
Medical Aid	6.3	12.8	2.1
1st (poorest)	7.1	11.6	3.6
2nd	4.2	19.7	0.5
3rd	3.4	22.9	0.8
4th	2.2	52.1	1.2
5th (richest)	3.1	54.1	0.6

**Table 4.** Comparative analysis of deaths among long-term hospitalized patients after the closure of Jinju Medical Center

Variables	Comparison of mortality rates by hospital type				
	No. of long-term inpatients (A)	No. of deaths within 1 mo (B)	Ratio (A/B)	No of deaths within 1 yr (C)	Ratio (A/C)
Jinju Medical Center	259	12	4.6	61	23.6
Other public hospitals	6,488	133	2.0	1,109	17.1
Private hospitals located in Jinju^1^					
Group 1	667	14	2.1	121	18.1
Group 2	2,108	30	1.4	291	13.8

1 Group 1: 22 Private hospitals located within Jinju City; Group 2: 3 Private hospitals with a similar number of beds to Jinju Medical Center.

**Table 5.** Comparative analysis of the probability of death of long-term hospitalized patients^1^

Characteristics		No. of patients	No. of deaths	OR (95% CI)	p-value
Hospital type	Private hospitals^2^	263	41	1.00 (reference)	
	Jinju Medical Center	247	49	2.47 (0.85, 0.96)	<0.001
	Other public hospitals	372	65	1.06 (0.91, 1.03)	0.148
	Other private hospitals^3^	584	103	1.14 (0.80, 0.90)	<0.001
Gender	Men	616	130	1.00 (reference)	
	Women	850	128	0.49 (0.48, 0.49)	<0.001
Age (yr)	≥80	656	152	1.00 (reference)	
	0-9	0	0	0.00 (0.00, 0.00)	<0.001
	10-19	0	0	0.00 (0.00, 0.00)	<0.001
	20-29	6	1	0.01 (0.01, 0.01)	<0.001
	30-39	12	2	0.01 (0.01, 0.01)	<0.001
	40-49	79	8	0.02 (0.02, 0.03)	<0.001
	50-59	152	17	0.05 (0.05, 0.05)	<0.001
	60-69	119	10	0.10 (0.10, 0.10)	<0.001
	70-79	442	68	0.33 (0.32, 0.33)	<0.001
Income	5th richest	526	72	1.00 (reference)	
	Medical Aid	362	87	2.24 (2.20, 2.28)	<0.001
	1st poorest	176	32	1.34 (1.32, 1.37)	<0.001
	2nd	120	21	1.28 (1.26, 1.32)	<0.001
	3rd	130	20	1.15 (1.13, 1.18)	<0.001
	4th	152	26	1.06 (1.04, 1.09)	<0.001
CCI	0	324	54	1.00 (reference)	
	1	542	99	1.06 (1.06, 1.06)	<0.001
	2	454	78	1.07 (1.07, 1.07)	<0.001
	≥3	146	27	1.09 (1.08, 1.09)	<0.001

OR, odds ratio; CI, confidence interval; CCI, Charlson comorbidity index.

1 Number of observations 1,466; Somers’ D: 0.767; C-statistic: 0.884.

2 Private hospitals located within Jinju City.

3 Private hospitals located in Gyeongsangnam Province (excluding Jinju).
